# The assessment of xenogeneic bone immunotoxicity and risk management study

**DOI:** 10.1186/s12938-019-0729-z

**Published:** 2019-11-14

**Authors:** Xiaoxia Sun, Chenghu Liu, Yanping Shi, Chunling Li, Likui Sun, Li Hou, Xin Wang

**Affiliations:** 1 Shandong Key Laboratory of Biological Evaluation for Medical Devices, Jinan, 250101 People’s Republic of China; 2 Shandong Quality Inspection Center for Medical Devices, No. 15166 Century Avenue, Jinan H-T Industrial Development Zone, Jinan, 250101 Shandong People’s Republic of China

**Keywords:** Xenogeneic bone, Immunotoxicity, Immune safety, Risk management

## Abstract

**Background:**

Xenogeneic bone has been widely used in a variety of clinical bone-related disease to promote bone healing and restore bone defects. However, the adverse effects of immune system limit its application in the clinic. The aim of this study was to evaluate xenogeneic bone safety of immunotoxicity and explore the methods for immune risk supervision.

**Results:**

Xenogeneic bone, which is freeze-dried bovine cancellous bone, was implanted into the muscle of mice. On day 7, 14 and 28, the effects of xenogeneic bone were examined on humoral immunity and cellular immunity, including the levels of IgG, IgM, C3, inflammatory factors (TNF-α, IL-6), alkaline phosphatase (ALP) and the lymphocyte phenotype. The data showed that xenogeneic bone implantation had no potential to induce immune responses not only in humoral immunity but also in cellular immunity. To reveal the risk of immunogenicity, the residual DNA and the clearance of α-gal epitope were analyzed in 2 different bones (bone 1 is deproteinized bone, bone 2 is acellular and defatted bone). It was suggested that DNA of xenogeneic bone can be limited to < 50 ng per mg dry weight for the repair or regeneration with the acceptable immune risk. And α-gal clearance of xenogeneic bone could be an effective risk factor for improving xenograft quality management.

**Conclusions:**

Through the detection of xenogeneic bone immunotoxicity, our findings indicated that the supervisions of risk factors could contribute to reduce the immune risk. And the risk factors under the acceptable limitation could decrease or replace animal experiment. However, it still needs to be studied on the limitation of α-gal epitope to predict rejection of xenogeneic bone more accurately.

## Background

Bone grafting, as a common therapeutic method for bone defects, can be classified into autogenic, allogeneic, xenogeneic grafting and synthetic bone based on the sources of the implant materials. Although autogenic bone is the first choice used as a bone grafting material [1, 2], its application is limited due to the donor bone shortage, donor area dysfunction. Allograft application was limited by the transfer of diseases. Xenogeneic bone, which has a variety of sources and the ability of osteoinduction and osteoconduction activities, could satisfy the requirements of ideal bone graft substitutes. However, the immune risks of xenogeneic bone, which affect the safety and effectiveness of the material, limit its application [3, 4]. Therefore, it is necessary to determine the safety of xenogeneic bone on the immune system.

The safety evaluation has two parts, immunotoxicity assessment and risk management on the immunogenicity. Safety evaluation, which means to predict the adverse reactions of recipient’s immune system, is essential to improve engraftment rates. The potential immunotoxicity of xenogeneic bone might include inflammation, immunosuppression, immunostimulation and hypersensitivity. Although there is an accepted standard for the immunotoxicity testing (ISO/TS 10993-20: 2006), methods for the detection may be varied due to xenografts’ properties, such as their derivation, processing and application [5, 6]. These properties can be seemed as hazards related to the immunotoxicity of xenografts. Thus, it is vital for the identification and management of risks so as to minimize the risk of immunotoxicity.

Immune responses, between the antigen on xenogeneic bone and the antibody in human, may lead to a precocious re-absorption, fibrosis of the implant, implant rejection, and eventually failure of the intervention [1, 7]. Antigens, including MHC and α-gal epitope, may exist in the xenogeneic scaffolds that have not been properly decellularized and can be carried by osteocytes, osteoblasts, osteoclasts and bone marrow cells [4, 8]. Studies have shown that deproteinized bone not only lose their immune reactivity but also retain their osteoinduction and osteoconduction activities [9]. And other types of xenogeneic bone are available: decalcified bone, freeze-dried bone and defatted bone [2]. Prior to the immunotoxicity assessment, the immune risk supervision of xenogeneic bone can contribute to reduce immune responses, promote the commercial bone development and application. However, there is still lack of the established criteria for the risk management of xenogeneic bone.

This study focuses on immune toxicity of xenogeneic bone and tries to assess its safety by the means of simulating clinical use. Xenogeneic bone used in this study is freeze-dried bovine cancellous bone scaffolds (bone 1 is deproteinized bone, bone 2 is acellular and defatted bone). The effects on humoral immunity and cellular immunity were analyzed to illustrate its immune toxicity using the proliferation of lymphocyte test and muscle implant experiments; In addition, the residual DNA and the clearance of α-galactosidase (α-Gal) epitope were determined and could be used as the risk factors to supervise the immune risk for xenogeneic bone application.

## Results

### Extracts of xenogeneic bone have no effect on human peripheral blood mononuclear cell (hPBMC) proliferation

**Fig. 1:**
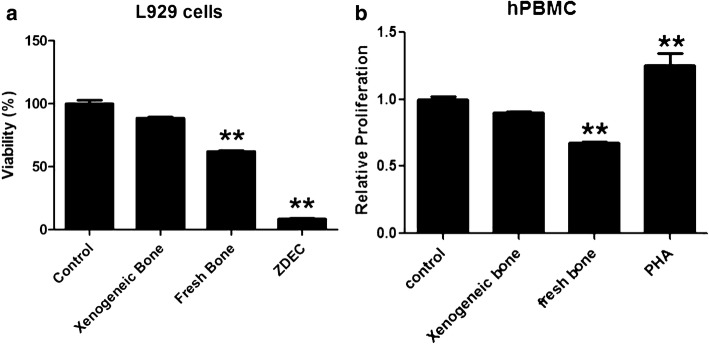
Xenogeneic bones have no effect on cells proliferation. The test solutions of xenogeneic bone (bone 1), fresh bone and ZDEC are prepared according to ISO10993-12: 2012. The proliferation of test solutions on L929 (**a**) and hPBMC (**b**) was detected by MTT assay. RPMI medium 1640 was used as control, ZDEC was used as positive control in L929 test, and PHA (10 μg/mL) was used as positive control in hPBMC test. The histogram indicated the viability of L929 or relative proliferation of PBMC using the following formula: viability (%) = optical density value of test sample/optical density value of control × 100; relative proliferation = optical density value of test sample/optical density value of control. Data were representative of at least three independent experiments; statistical significance was defined as **p < 0.01 and *p < 0.05 compared with control

L929 cell lines, mouse fibroblast cells, are commonly used to evaluate the cytotoxicity of medical devices in ISO 10993-5: 2009. According to sample preparation in ISO10993-12: 2012 and MTT test, L929 cells were cultured with the solutions extracted from test samples for 72 h. The result showed that the growth of L929 was inhibited by the extracts of fresh bone, while the extracts of xenogeneic bone (bone 1) had no effect on L929 proliferation (Fig. 1a). The viability in control was used as 1, and viabilities in xenogeneic bone, fresh bone and ZDEC group were 0.885 ± 0.022, 0.620 ± 0.019, 0.093 ± 0.017, respectively.

Xenografts with biological activity may trigger immune responses during clinical use. Therefore, the proliferation of hPBMC in vitro was measured to verify the response of lymphocyte to xenogeneic bone (bone 1). As shown in Fig. 1b, compared with control, the extracts of xenogeneic bone had no effect on the proliferation of hPBMC (relative proliferation: 0.903 ± 0.018, *p* > 0.05), while extracts of fresh bone have an obvious inhibitory effect on hPBMC (relative proliferation: 0.678 ± 0.016, *p* < 0.01). The results of cytotoxicity test and hPBMC proliferation indicated that there was no immune response to xenogeneic bone in vitro.

### Implantation of xenogeneic bone had no effect on serum IgG, IgM and Complement 3 (C3) level

**Fig. 2:**
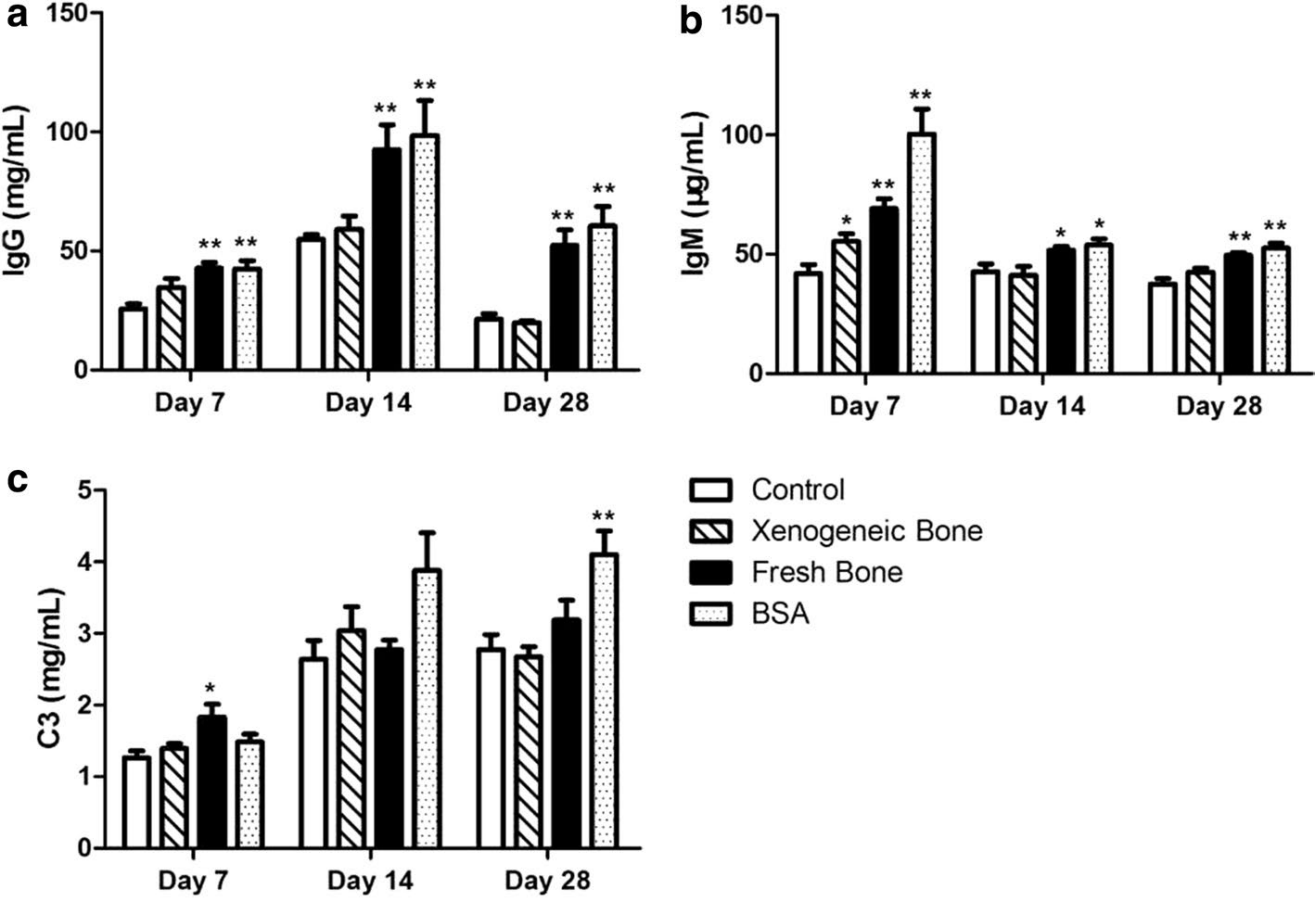
Detection of immunoglobin and complement in xenograft mice. Xenogeneic bone (bone 1) was implanted into Balb/c mice, the levels of IgG (**a**), IgM (**b**) and C3 (**c**) in the serum were measured by ELISAs on day 7, 14 and 28. The sham operation was used as control. Statistical significance was defined as **p < 0.01 and *p < 0.05 compared with control

Under the guidance of ISO/TS 10993-20: 2006, the immune response of xenogeneic bone (bone 1) was examined by means of muscle embedding into Balb/c mice. The sham operation was used as control, the positive control is BSA treatment and the implantation of fresh bovine bone was used as raw material control group. The mean body weight of mice increased gradually during the whole experiment (Additional file 1: Figure S1), and total serum IgG and IgM levels were measured by ELISA assay. As shown in Fig. 2a, b, except for transient increase of IgM (*p* < 0.05) after xenogeneic bone implantation on day 7, there was no differences in the levels of IgG (19.921 ± 1.938 mg/mL compared with 21.320 ± 6.389 mg/mL of control on day 28, *p* > 0.05) and IgM (42.495 ± 4.603 μg/mL compared with 37.444 ± 6.160 μg/mL of control on day 28, *p* > 0.05) between xenogeneic bone and control (*p* > 0.05). While IgG and IgM levels of fresh bone and BSA group were significantly higher than those of control groups on day 7, 14 and 28 (*p* < 0.01).

Compared to control, no differences were found on the level of C3 within the experimental period of xenogeneic bone implantation (2.673 ± 0.376 mg/mL compared with 2.775 ± 0.554 mg/mL of control on day 28, *p* > 0.05), while C3 of BSA group were much higher on day 28 (Fig. 2c, *p* < 0.01). The aberrant increased IgG, IgM and C3 levels suggested that the implantation of fresh bone, not xenogeneic bone, could mediate inflammation and immunotoxicity response.

### Xenogeneic bone implantation could not cause inflammatory reaction

**Fig. 3:**
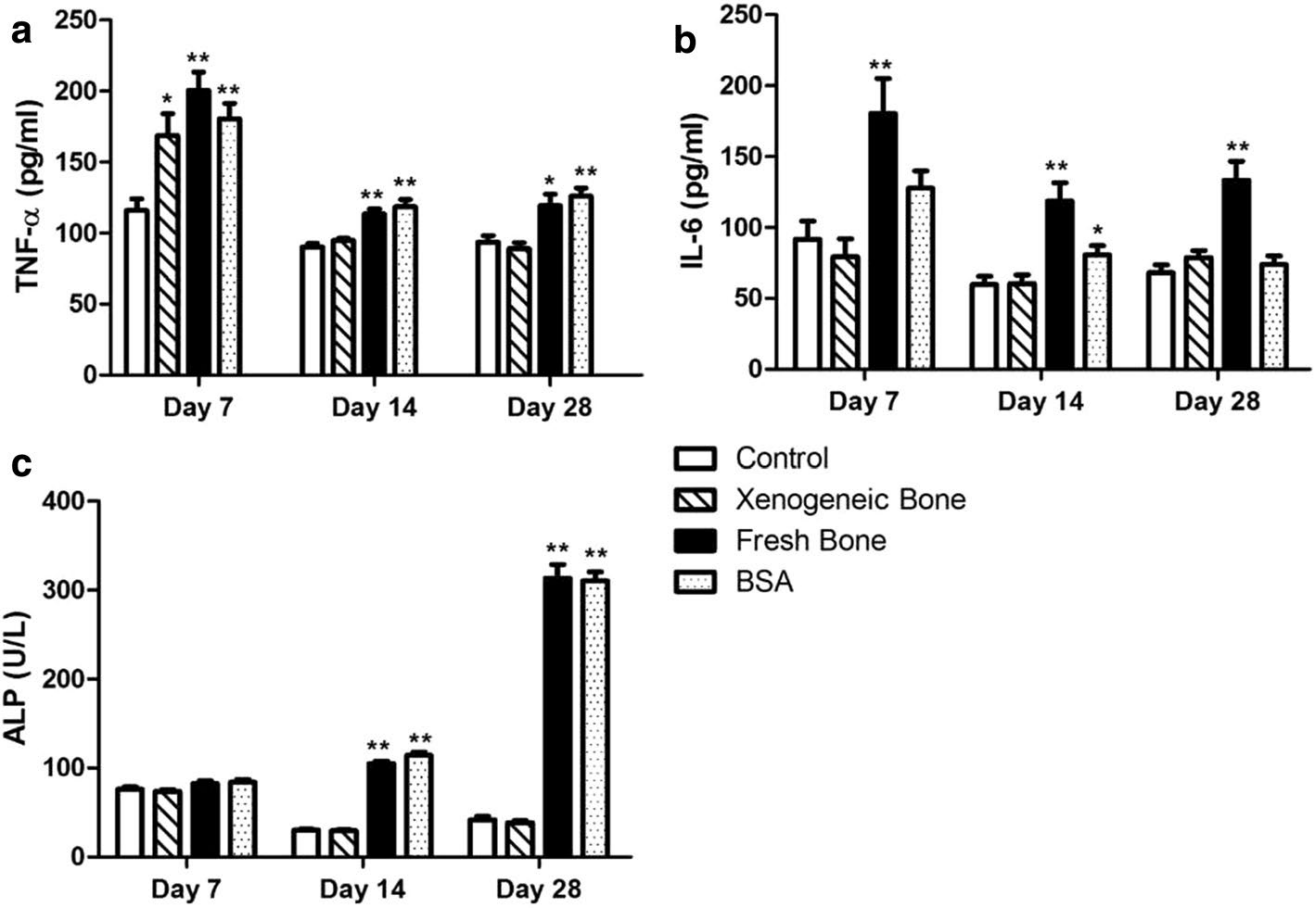
Inflammatory factors had no change after xenogeneic bone implantation. Xenogeneic bone being (bone 1) was implanted into Balb/c mice, TNF-α (**a**) and IL-6 (**b**) levels were measure using ELISAs and muscle ALP activities (**c**) in the site of implantation are quantified by colorimetric assay on days 7, 14 and 28. Statistical significance is defined as **p < 0.01 and *p < 0.05 compared with control

To further clarify the inflammatory response, the inflammatory factors (TNF-α and IL-6) after implantation were measured by ELISA assay. The results showed that, compared to control groups on day 7, 14 and 28, the implantation of xenogeneic bone (bone 1) had no effect on the serum TNF-α (88.981 ± 11.817 pg/mL compared with 93.621 ± 13.068 pg/mL of control on day 28, *p* > 0.05) and IL-6 (78.737 ± 13.750 pg/mL compared with 67.922 ± 16.059 pg/mL of control on day 28, *p* > 0.05) levels except for a transient increase of TNF-α on day 7 (Fig. 3a, b, *p* < 0.05); while the treatment of fresh bone and BSA could keep the increase of TNF-α and IL-6 (*p* < 0.01), which were in accordance with C3 level and indicated the continued development of inflammation.

Additionally, the local inflammatory response was examined. ALP is not only closely related with the process of bone formation, but also involved in the pathological process of liver and kidney disorders, inflammation and tumor [10]. Muscle ALP activities in the site of implantation were measured by colorimetric assay on day 7, 14 and 28 after xenotransplantation. As shown in Fig. 3c, similar to the results of serum inflammatory factors, there is no significant changes on muscle ALP activity between xenogeneic bone implantation (38.374 ± 7.062 U/L on day 28) and control (41.890 ± 11.177 U/L on day 28) on each time point. But ALP activity was upregulated obviously, even up to 300 U/L, by fresh bone (*p* < 0.01) and BSA treatments (*p* < 0.01). The data of inflammatory factors confirmed that xenogeneic bone could not induce inflammation, while fresh bone and BSA treatment could promote the inflammatory response which was positively related to the activation of complement system.

### Implantation of xenogeneic bone had no effect on the lymphocyte subset distribution

**Fig. 4:**
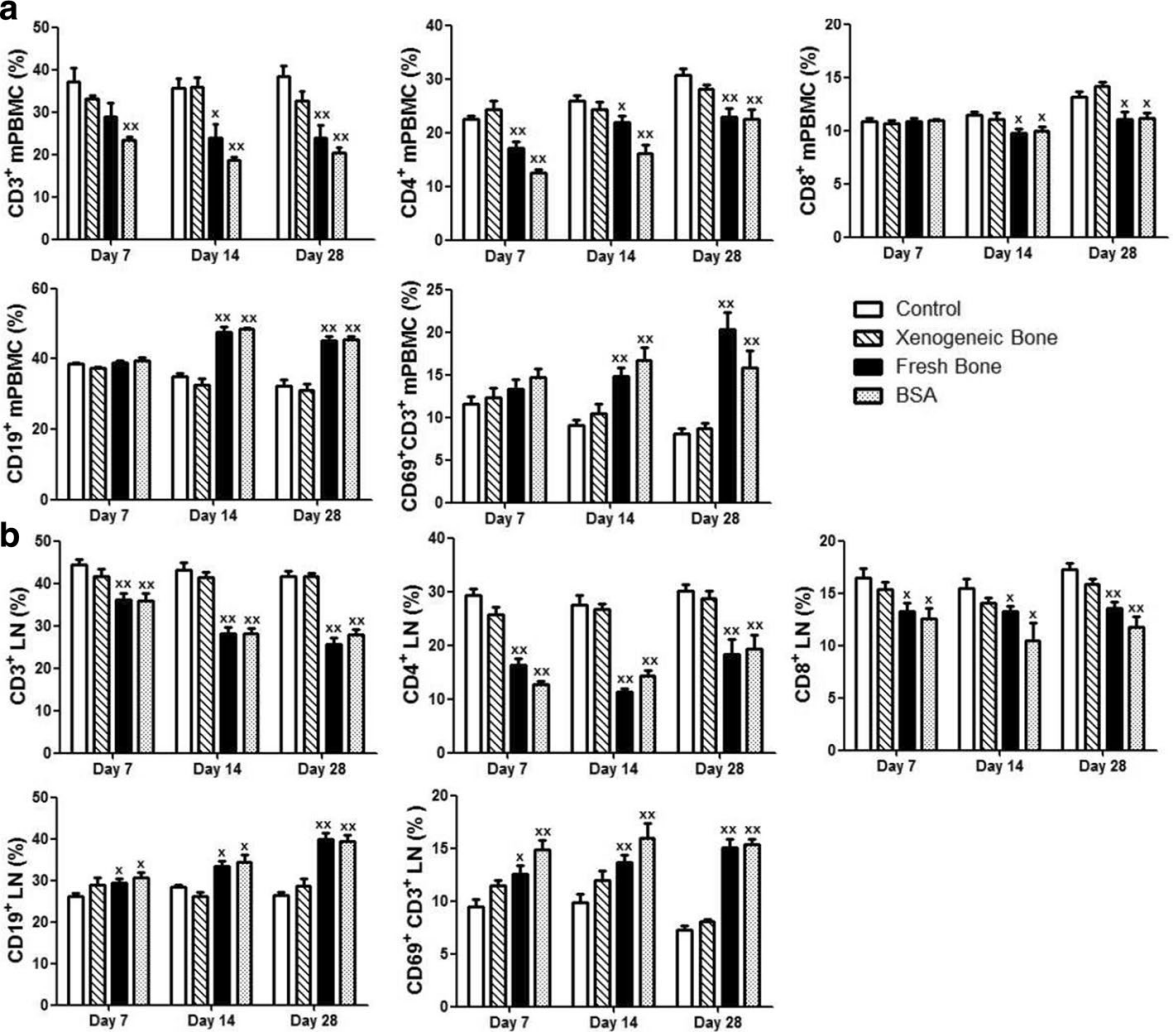
Xenogeneic bone has no effect on the phenotype of mouse lymphocytes. The lymphocytes, including mPBMC (**a**) and LN lymphocyte (**b**), were isolated from Balb/c mice and analyzed by flow cytometry. The histogram represented statistical analysis of the percentage positive cells. Statistical significance was determined as **p < 0.01 and *p < 0.05 compared with control

As one of the main types of immune cell, lymphocytes play important roles in both humoral and cellular immune responses. Mice peripheral blood mononuclear cells (mPBMC) were isolated and identified by flow cytometry assay. As shown in Fig. 4a, CD3^+^ (*p* < 0.01), CD4^+^ (*p* < 0.01) and CD8^+^mPBMCs (*p* < 0.05) were lower and CD3^+^CD69^+^mPBMCs (*p* < 0.01) were higher in fresh bone and BSA group than those cells in control group on day 28. In addition, fresh bone and BSA treatment could increase the proportion of B lymphocyte (CD19^+^mPBMC) obviously (*p* < 0.01), which was consistent with the high level of serum IgG and IgM. There were no differences on the proportions of CD19^+^mPBMCs between xenogeneic bone group (31.204 ± 4.470% on day 28) and control (32.420 ± 4.474% on day 28). However, beside B lymphocyte, the proportions of T lymphocytes [CD3^+^ (32.724 ± 4.898% on day 28), CD4^+^ (28.230 ± 2.343% on day 28), CD8^+^ (14.160 ± 1.313% on day 28) and CD3^+^CD69^+^ (8.736 ± 1.622% on day 28) mPBMCs] had no difference between xenogeneic bone group and control. These findings showed that the implantation of xenogeneic bone could not trigger humoral and cellular immune responses.

Furthermore, the draining lymph node (LN) lymphocytes of the implant site were isolated to examine the local immunotoxicity induced by xenogeneic bone. As shown in Fig. 4b, xenogeneic bone had no effect on both T cells and B cells of the draining LN which is similar to its findings of mPBMC. While compared to the control, significance changes had been found on the T and B lymphocytes of draining LN in fresh bone group and BSA group. The results of immune response from the systemic to the local suggested xenogeneic bone could not lead to immunotoxicity on Balb/c mice.

### Residual DNA of xenogeneic bone

**Fig. 5:**
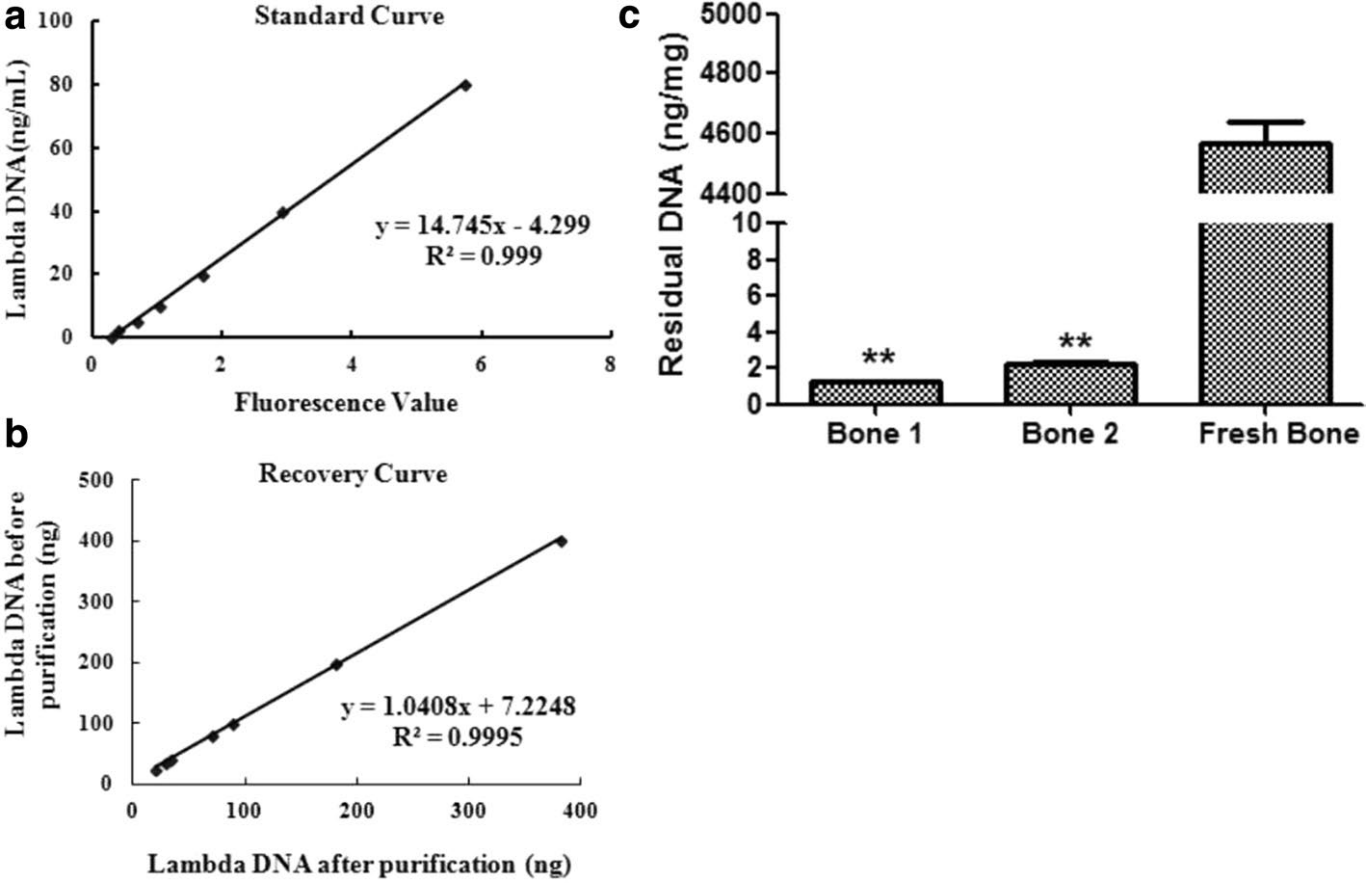
Residual DNA of xenogeneic biological bone was much lower than fresh bone. The residual DNA assay was performed to determine the DNA of xenogeneic bone per unit mass. **a** The standard curve was drawn with Lambda DNA (standard DNA) and fluorescence values, **b** the recovery curve was made based on pre- and post purification of Lambda DNA. And the residual DNA of xenogeneic bone per unit mass was calculated using standard curve and recovery curve (**c**). Statistical significance was determined as **p < 0.01 and *p < 0.05 compared with fresh bone

For biologically derived materials, quantification of residue DNA has been an effective method to control the medical products safety and supervise the risk of immunotoxicity [11]. In this study, DNA was isolated based on the principle of magnetic particles binding DNA, then the residue DNA was determined using Quant-iT PicoGreen assay. However, DNA quantification might be influenced by the operation error such as binding with magnetic particles, washing and elution. So it is necessary to set recovery curve to revise residue DNA by measuring DNA of pre- and post purification. In combination with standard curve (Fig. 5a) and recovery curve (Fig. 5b), the accurate residue DNA could be acquired. As shown in Fig. 5c, residue DNA from 2 commercial bones were both much lower than fresh bone (*p* < 0.01), and the DNA amount was different although they are from the same species.

### Clearance of α-gal epitope about xenogeneic bone

**Fig. 6:**
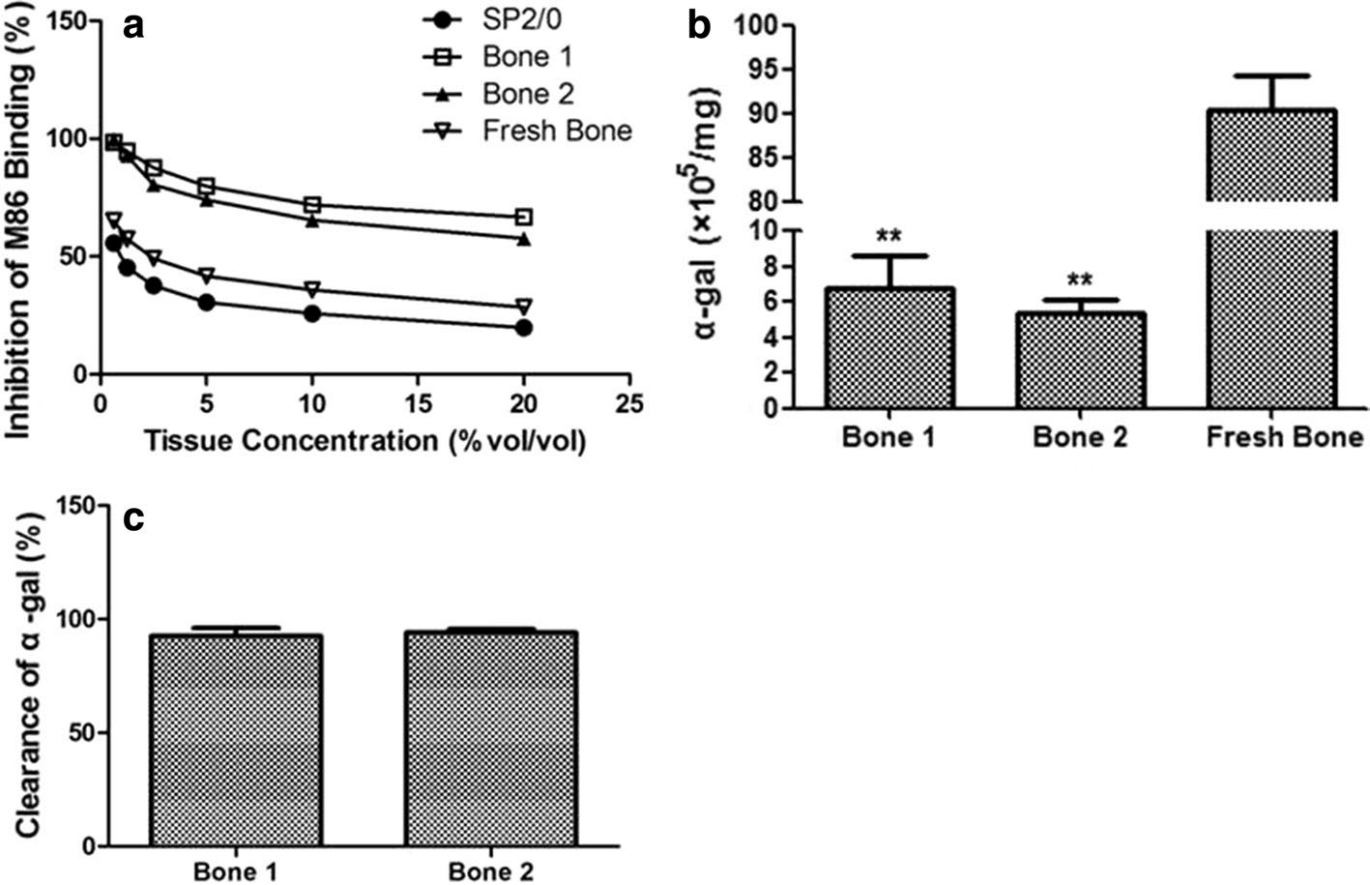
Determination of α-gal epitope in xenogeneic bone. ELISA inhibition assay was tested with xenogeneic bone, fresh bone and SP2/0 cells. **a** The concentration of tissues homogenates were expressed as  % (vol/vol). **b** α-gal epitope in xenogeneic bone and fresh bone were determined with SP2/0 cells as a standard. **c** The clearance of α-gal was calculated compared with fresh bone. Statistical significance was determined as **p < 0.01 and *p < 0.05 compared with fresh bone

Galactose-alpha-1,3-galactose, commonly known as α-gal, is a carbohydrate found in most organisms’ cell membranes. But it is not found in primates including humans, whose immune systems recognize it as a foreign body and produce xenoreactive antibodies, leading to immune rejection after transplantation [12]. It has been reported that the expression of α-gal could be determined by an ELISA inhibition assay, in which the interaction of M86 (monoclonal anti-gal antibody) with α-gal epitope on cells is measured by the activity of free M86 left in the supernatant [12]. And the extent of α-Gal expression on the xenogeneic bone correlates with the subsequent inhibition of M86 binding in ELISA. Studies have shown that there were approximately 10^6^ α-gal epitopes in each mouse myeloma SP2/0 cell which can be used as a standard in the ELISA assay [13]. To avoid the operation error, such as antigen coating and the affinity between residue M86 and α-gal epitope, it is useful to evaluate the α-gal clearance followed by the analysis of the difference of α-gal between fresh bone and xenogeneic bone (Fig. 6a). The results showed that α-gal epitopes on the xenogeneic bones were obviously decreased compared to fresh bone (Fig. 6b) with the clearance of α-gal were 92.5% (bone 1) and 94.0% (bone 2) (Fig. 6c).

## Discussion

Xenogeneic bone has been commonly use as a substitute for bone defect with the good biocompatibility and good biological safety [14, 15]. And many studies have been conducted to improve their poor integration compared to autografts and allografts, such as reconstruction xenograft. However, the application of xenogeneic bone is limited due to the immune rejection induced by antigen [3]. This study focuses on the immune safety evaluation of xenogeneic bone so as to improve engraftment rate.

To investigate the immunotoxicity, xenogeneic bone (bone 1) was implanted into the intermuscular space of mice similar with the clinical application. Balb/c and C57BL/6 mice are two common inbred strains used frequently in research. Balb/c mice are more susceptible to viral infections while exhibiting decreased cytotoxic responses but increased humoral or immunoglobulin responses to allergens and infections. In contrast, C57BL/6 mice are more resistant to viral infection exhibiting increased cytotoxic responses and decreased humoral and allergic responses. The lack of a strong protective response in Balb/c has been associated with a reduced ability to express the Th1 cytokine (IFN-γ and IL-12). Whereas resistant C57BL/6 mice preferentially activate Th1 cells [16, 17]. Therefore, female and male Balb/c mice were employed in this study. At first, two kinds of assays were examined to evaluate the immunotoxicity of xenogeneic bone: functional and non-functional. Functional assays determine activities of cells and/or organs, such as proliferative responses of lymphocytes to mitogens or specific antigens, cytotoxic activity and specific antibody formation. As shown in Fig. 1, xenogeneic bone had no effect on the proliferation of human PBMC. On the other hand, non-functional assays have a descriptive character in that they measure, in morphological and/or quantitative terms, the number of lymphoid cell and levels of immunoglobulins or other markers of immune function. Compared with the control, there was no obvious changes on serum levels of C3, IgG, IgM, inflammatory factors (TNF-α, IL-6) and the proportion of PBMC subsets after xenogeneic bone implantation (Figs. 2, 3, 4). Complement system is a physiological constituent of blood plasma and an important part of immune system, which involves a number of autoimmune and inflammatory diseases [18–20]. C3, the highest complement component in serum, plays a central role in the activation of immune system [21]. Moreover, there were no changes of inflammatory factors (TNF-α, IL-6) between xenogeneic bone implantation and control group (sham operation). These results indicate that there has no potential of immunotoxicity after xenogeneic bone implantation.

Beside systemic reactions, the experiments are designed to determine the local immune response to the xenogeneic bone [10, 22]. As shown in Fig. 3b, c, there was no difference on ALP activities and LN lymphocytes between xenogeneic bone and the control. These findings showed that xenogeneic bone has no potential of immunotoxicity not only in systemic but also in local immune reactions. Whereas compared to the control, great changes have been found in the above immunotoxicity assay after fresh bone and BSA treatment. And immune responses can be triggered both by the antigens of fresh bone and by BSA. It is suggested that BSA treatment can be used as the positive control for the immunotoxicity assay.

Many immune responses have been induced by xenografts application, including immunosuppression, immunostimulation, hypersensitivity, chronic inflammation and autoimmunity [21–24]. However, it is difficult to predict and evaluate the immunotoxicity of biological materials sufficiently. Then the additional efforts are needed to evaluate and manage the potential risks arising from immunogenic component of xenografts. The residual material within xenograft may be recognized by human immune system and result in adverse immune response [3, 25]. Therefore, it is necessary to determine the immunogens so as to evaluate and supervise the immunological hazard of xenograft.

The immunogens within xenograft comprise of the epitope of cytomembrane, heterogeneity DNA and small molecular substances. Many techniques have been employed to reduce or avoid the antigenicity, such as frozen, deproteinization, decalcification and lipophilization [9, 18, 26]. Although none of them have so far gained widespread acceptance, it is possible to quantitatively assay cell components such as double-stranded DNA (dsDNA). Xenogeneic bones used in this study have different with treatments, one is deproteinized and freeze-dried bone (bone 1) and another is acellular, defatted and freeze-dried bone (bone 2). While residue DNA assay showed that DNA of 2 commercial xenogeneic bones were both much lower than fresh bone, which were 1.3 ng and 2.3 ng per mg of dry weight, respectively (Fig. 5). The studies on extracellular matrix (ECM) have shown that host responses should be avoided during the process of constructive remodeling. Based on these findings, the acceptable criteria for DNA suffice to satisfy the intent of immunogens minimization: < 50 ng DNA per mg ECM dry weight [27, 28]. Although commercially xenogeneic bone can be very different in not only bone source but also tissue type into which it is implanted, DNA of xenogeneic bone can be limited to < 50 ng per mg dry weight with the aim of repair or regeneration. Therefore, it is essential for xenogeneic bone to use the residual DNA as a risk factor for the supervision of adverse immune responses.

In addition, α-Gal epitope, as a non-self-antigen, has been shown to elicit immune rejection by stimulating anti-α-Gal antibodies and might be another immune factor for risk management [8]. However, the α-gal epitope expressions on various cells are different [29, 30]. And it is well-known that beside α-gal epitope, other antigens (e.g., swine leukocyte antigen) are also responsible for graft rejection [8, 31]. Thus, it is difficult to limit the amount of α-gal epitope in xenograft. The results of our study showed that α-gal clearance of xenogeneic bone could contribute to supervise product quality (Fig. 6 and Additional file 2: Figure S2). Although several studies have focused on the elimination of α-gal epitope from donor tissue, the outcome assessment was not believed to be well established [3, 32]. Thus, further study on the determination of specific antibody induced by xenograft, such as the IgM and IgG anti-α-gal antibody, will contribute to find out the limitation of α-gal epitope and predict rejection of xenogeneic bone.

## Conclusions

In this study, we focus on the immunotoxicity of xenogeneic bone and the risk factors related with immunotoxicity. The results show that there is no immunotoxicity after the implantation of xenogeneic bone. While BSA treatment can induce humoral and cellular immune response in Balb/c mice which can also be found after fresh bone treatment. After the analysis of the immunogens of xenogeneic bone, it is suggested that residual DNA and α-Gal epitope can be used as risk factors to predict the likelihood of xenogeneic bone immunotoxicity. Supervising the risk factor under the acceptable limitation will contribute to reduce the immune risk, improve xenograft quality management and decrease the animal experiment.

## Methods

### Xenogeneic bone

Xenogeneic bones were generously provided by Guanhao Biotech (Bone 1: deproteinized bovine cancellous bone with freeze-drying, Guangzhou, Guangdong Province, China) and Zhenghai Biotech Co., Ltd. (Bone 2: acellular and defatted bovine cancellous bone with freeze-drying, Yantai, Shandong Province, China). The fresh bovine bone (i.e., fresh bovine bone) was used as material control.

### The proliferations of L929 and human peripheral blood mononuclear cells (hPBMC)

Based on the principle of ISO10993-12: 2012 (sample preparation and reference materials), xenogeneic bone (bone 1) was covered with RPMI medium 1640 (containing 10% FBS) at the proportion of 0.1 g/mL with adding additional material absorbs. The test solution of xenogeneic bone was prepared at the condition of (37 ± 1) °C for (24 ± 2)h. The same method was employed to treat fresh bone and ZDEC (RM-A, polyurethane film containing 0.1% zinc diethyldithiocarbamate, purchased from Hatano Research Institute, Japan).

For the cell proliferation assay, MTT assay was used according to ISO10993-5: 2009 (Tests for in vitro cytotoxicity). L929 cells (ATCC, CCL-1™) or primary human peripheral blood mononuclear cells (hPBMC) were resuspended with the test solutions, respectively. Seed cells into 96-well and incubate for (72 ± 2)h in a incubator (37 °C, humidified, 5% CO_2_/air). During the last 4 h of incubation, MTT (Sigma) was present in the culture. Then isopropanol (Sigma) was added to dissolve the formazan crystals and the absorbance was read at a wavelength of 570–630 nm using a Microplate Autoreader (Molecular Devices). hPBMC were freshly isolated by Ficoll density gradient centrifugation and cultured in RPMI-1640, containing 10% FBS and 100 U/mL rhIL-2. Informed consent was provided by all participants enrolled in this study. RPMI medium 1640 was used as control. Material reference control (ZDEC, polyurethane film containing 0.1% zinc diethyldithiocarbamate, Hatano Research Institute) was used as positive control in L929 test, and phytohemagglutinin (PHA, 10 μg/mL, Sigma-Aldrich, St Louis, USA) was used as positive control in hPBMC test.

### Implantation of xenogeneic bone into Balb/c mice

The animals were anesthetized by intraperitoneal route with 55 mg/kg (3% diluted in 0.9% NaCl) of pentobarbital sodium. To mimic the clinical use, xenogeneic bone (bone 1) was prepared into small pieces (3 mm × 3 mm × 3 mm) and was embedded into the intermuscular space of 6-week-old Balb/c mice right thigh.

Besides of xenogeneic bone group, there were three other groups employed in our study, that is: (a), raw material group, which was fresh bovine bone with the same size as xenogeneic bone (3 mm × 3 mm × 3 mm). It was used as the positive control material; (b), control group, that was sham operation to eliminate the interference of operation; (c), BSA (bovine serum albumin, Sigma) group, which was used as positive control to verify the immune reaction of immune stimulus on Balb/c mice. 3 mL of solution which were prepared with 3 mg of BSA dissolved in 9 mL of PBS (PH = 7.4), were mixed with 3 mL Freund’s adjuvant thoroughly, then 0.12 mL of mixture was administered intraperitonealy once a week. No less than 6 animals each group with half male and half female. All procedures were performed in accordance with Institutional Animal Care and Use Committee Protocols.

### Enzyme-linked immunosorbent assay (ELISA)

IgG, IgM, C3 (Alpha Diagnostic International, TX, USA), IL-6 and TNF-α (Invitrogen Co., CA, USA) levels in the mouse serum were determined using ELISA kits according to each manufacturer’s instructions.

### Alkaline phosphatase (ALP) activity measurement

Muscle ALP activities in the site of implantation were quantified by colorimetric assay (Zhicheng Biological Technology Co., Shanghai, China) on day 7, 14 and 28 after xenotransplantation. Samples, standards and reagents were all used according to manufacturer’s instructions.

### Isolation of mouse lymphocytes

Mice peripheral blood mononuclear cell (mPBMC) was isolated by Ficoll density gradient centrifugation and cultured in RPMI-1640, containing 10% FBS and 100 U/mL rhIL-2.

To isolate lymphocytes from the axillary and inguinal lymph nodes (LNs), mouse axillary and inguinal LNs were removed and pressed separately through a 200-gauge stainless steel mesh, and the cell suspension was collected after washing with PBS.

### Flow cytometry

For the phenotype assay, cells were incubated with fluorescence-conjugated antibodies for 30 min at 4 °C. Subsequently, washing unconjugated Abs with PBS, and then stained cells were acquired using FACSCalibur system (BD Biosciences, San Jose, CA, USA) and analyzed with WinMDI 2.0 software. The fluorescence-conjugated antibodies are described in Additional file 3: Table S1.

### Residual DNA assay

The xenogeneic bone was weighed in the aseptic condition and was digested by proteinase K in DNase-free sterile centrifuge at 56 °C water bath. Then DNA were extracted and purified with PrepSEQ™ Residual DNA Sample Preparation Kit (Life technologies, Warrington, UK), and fluorescence values were measured with fluorescence microplate reader according to the protocol of Quant-iT™ PicoGreen™ dsDNA Assay Kit (Life technologies, Oregon, USA). At the meanwhile, Lambda DNA (standard DNA) was selected to make standard curve and pre- and post purification of Lambda DNA (standard DNA) were measured to perform recovery curve. Finally, the DNA of the xenogeneic bone per unit mass could be calculated exactly followed by standard curve and recovery curve.

### Enzyme-linked immunosorbent assay (ELISA) inhibition test for α-gal epitope

The xenogeneic bone was ground for two or more times into powder in the low-temperature homogenizer. The weighed bone powder mixed with 1% BSA to form tissue homogenate at the aseptic condition. Then the homogenate was incubated overnight with the monoclonal anti-Gal antibody (M86, purchased from Enzo Life Science, NY, USA). The homogenate precipitation and bound antibody were removed, and the residual antibody in the supernatant was measured in an ELISA assay with α-Gal-BSA (Dextra Laboratories, RG, UK) as a solid phase antigene. In addition to the xenogeneic bone group, fresh bovine bone, blank control (i.e., the test solvent control group) and the SP2/0 cell standard were simultaneously setup. The following formula was used to calculate α-Gal clearance. The inhibition of M86 binding (%) = the absorbance value of test article/the absorbance value of blank × 100; α-Gal antigen clearance (%) = (1-the concentration of test article at 50% inhibition/the concentration of fresh bone at 50% inhibition) × 100.

### Statistical analysis

All data are mean ± SD of three or more independent experiments or ≥ six animals. The data were analyzed by SPSS for statistical significance. Bartlett method for homogeneity was used to select the type of analysis to be conduct. Homogeneous data were analyzed using one-way analysis of variance, and multiple Dunnett’s Tests was used to determine differences between the control and experimental groups. Non-homogeneous data were evaluated using a non-parametric analysis of variance. When significant differences occurred, treatment groups were compared to control groups using the Wilcoxon Rank Test. Significance was defined as *p < 0.05 and **p < 0.01.

## Supplementary information

**Additional file 1: Figure S1.** Xenogeneic bones have no effect on the weights of xenograft mice. Animals were weighed on the day prior to treatment, on alternate weeks until day of sacrifice, and on day of sacrifice.

**Additional file 2: Figure S2.** The expressionof α-Gal could be decreased through decellularization. Images of sections of decellularized (A) and native (B) porcine dermal with antibody to α-Gal epitope (M86). The images captured at 10× magnification. Scales bars 200 μm.

**Additional file 3: Table S1.** Fluorochrome-conjugated antibodies used in flow cytometry.

## Data Availability

Data related to the current study are available from the corresponding author on reasonable request.

## Abbreviations

ALP: Alkaline phosphatase
hPBMC: Human peripheral blood mononuclear cells
mPBMC: Mice peripheral blood mononuclear cells
BSA: Bovine serum albumin
ELISA: Enzyme-linked immunosorbent assay
LN: Lymph node
dsDNA: Double-stranded DNA
ECM: Extracellular matrix
PHA: Phytohemagglutinin
