# Supplementary figures and images for: The assessment of xenogeneic bone immunotoxicity and risk management study
Source: Biomed Eng Online. 2019 Nov 14;18:108. doi: 10.1186/s12938-019-0729-z (PMC6857292; doi:10.1186/s12938-019-0729-z)

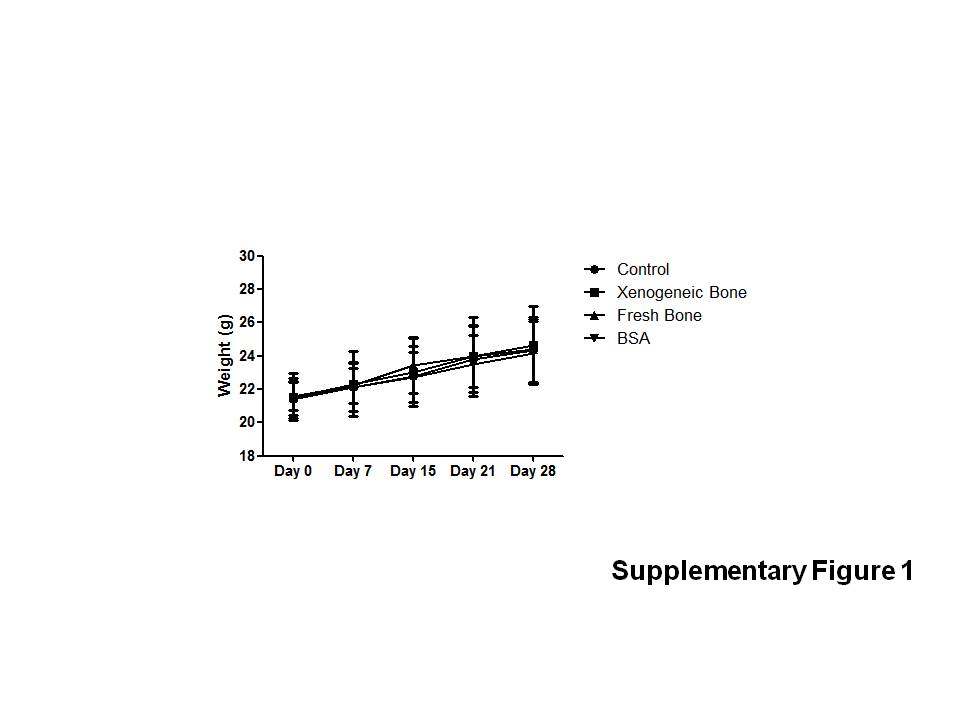

Supplement: Supplementary file 1 — Additional file 1: Figure S1. Xenogeneic bones have no effect on the weights of xenograft mice. Animals were weighed on the day prior to treatment, on alternate weeks until day of sacrifice, and on day of sacrifice. [file 12938_2019_729_MOESM1_ESM.jpg]

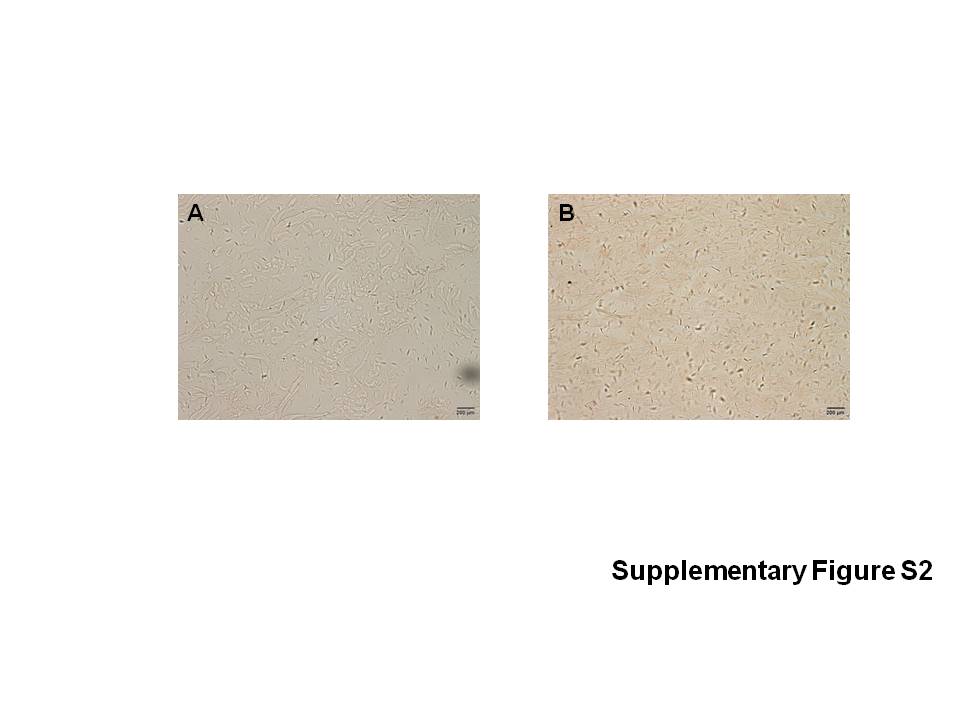

Supplement: Supplementary file 2 — Additional file 2: Figure S2. The expressionof α-Gal could be decreased through decellularization. Images of sections of decellularized (A) and native (B) porcine dermal with antibody to α-Gal epitope (M86). The images captured at 10× magnification. Scales bars 200 μm. [file 12938_2019_729_MOESM2_ESM.jpg]
