# Supplementary material for: The assessment of xenogeneic bone immunotoxicity and risk management study
Source: Biomed Eng Online. 2019 Nov 14;18:108. doi: 10.1186/s12938-019-0729-z (PMC6857292; doi:10.1186/s12938-019-0729-z)
Supplement: Supplementary file 3 — Additional file 3: Table S1. Fluorochrome-conjugated antibodies used in flow cytometry. [file 12938_2019_729_MOESM3_ESM.doc]

**Additional file 3: Table S**1. Fluorochrome-conjugated antibodies used in flow cytometry.

| Antibody | Producer |
| --- | --- |
| FITC anti-mouse CD3 antibody | BioLegend |
| FITC Rat IgG2b,κ Isotype Ctrl Antibody | BioLegend |
| PE anti-mouse CD8 antibody | BioLegend |
| PE Rat IgG2a,κ Isotype Ctrl Antibody | BioLegend |
| PE anti-mouse CD69 antibody | BioLegend |
| PE Armenian Hamster IgG Isotype Ctrl Antibody | BioLegend |
| PerCP anti-mouse CD45 antibody | BioLegend |
| APC anti-mouse CD4 antibody | BioLegend |
| APC Rat IgG2b,κ Isotype Ctrl Antibody | BioLegend |
| APC anti-mouse CD19 antibody | BioLegend |
| APC Rat IgG2a,κ Isotype Ctrl Antibody | BioLegend |
| APC anti-mouse CD49b antibody | BioLegend |

Suppliers: BioLegend (San Diego, CA)
